# Live Birth from the Transfer of a Severely Fragmented Embryo Observed by Morphokinetics

**DOI:** 10.1155/2018/2152918

**Published:** 2018-07-19

**Authors:** S. Lahav-Baratz, I. Blais, M. Koifman, D. Ishai, Z. Wiener-Megnazi, G. Peer, M. Dirnfeld

**Affiliations:** ^1^Obs/Gyn Department IVF Unit, Lady Davis Carmel Medical Center, Haifa, Israel; ^2^Ruth and Bruce Rappaport Faculty of Medicine, Technion-Israel Institute of Technology, Haifa, Israel

## Abstract

We report a live birth from a heavily fragmented embryo which continued cleavage to a fully expanded blastocyst. A 32-year-old patient underwent 2 IVF cycles without achieving pregnancy. In the first cycle, 2 embryos with fragmentation were transferred; in the second, all embryos were fragmented and no embryo transfer was performed. In a third cycle, 12 oocytes were retrieved and 11 of them were fertilized. On day 2, all 11 embryos started to unwind to fragments. By careful annotation, using the time-lapse EmbryoScope, we observed that one embryo continued division as expected, discarding all fragments aside. On day 5, this embryo showed promising annotation according to our lab model. The embryo was transferred into the uterus and resulted in the birth of a healthy baby at term. To our knowledge, this is the first case report assisted by EmbryoScope where a healthy baby was delivered from a fragmented embryo.

## 1. Introduction

Embryo selection for transfer is based on embryo scoring, which includes cell number, cleavage rate, and percentage of fragmentation and symmetry of cells [1]. Recently, time-lapse technology has been shown to contribute to embryo selection by annotation of the timing and pattern of cell division of embryos in the IVF laboratory [2, 3]. Time-lapse technology, used in combination with morphologic features, has been reported to improve IVF outcomes [4, 5]. Embryos reaching the blastocyst stage are less likely to be aneuploid, and implantation rates are higher [6]. Use of time-lapse monitoring and extended culture to the blastocyst stage therefore enables selection of a single best embryo. We report a patient in whom continuous monitoring up to day 5 in the EmbryoScope yielded a single embryo that was implanted and matured to a healthy baby.

## 2. Materials and Methods

Patient-informed consent was not requested since all patient data were deidentified. In the first cycle, the patient received 150 IU/ 8 days of recombinant FSH (Gonal F) and GnRH antagonist (Orgalutran 0.25 mg) followed by recombinant hCG (Ovitrelle 0.25 mg) 36 hours prior to egg retrieval. On the second IVF cycle, the patient received Corifollitropin alfa (ELONVA 100 IU) and after 7 days recombinant FSH (Puragon 125 IU) was added for 3 days. On the third cycle, Human Menopausal Gonadotropin (Menopur 225 IU) was administrated for 3 days and then Menopur 150 IU for 5 days. Fertilized oocytes were incubated in the EmbryoScope (time-lapse system). Similar culture conditions were employed in all 3 IVF cycles: continuous media (Life Global), 37°C, 6.0% CO2, and 5% O2. Images were taken every 15 minutes at 7 different focal planes for each embryo. Annotations were performed using embryo-viewer software.

## 3. Results

**Figure 1:**
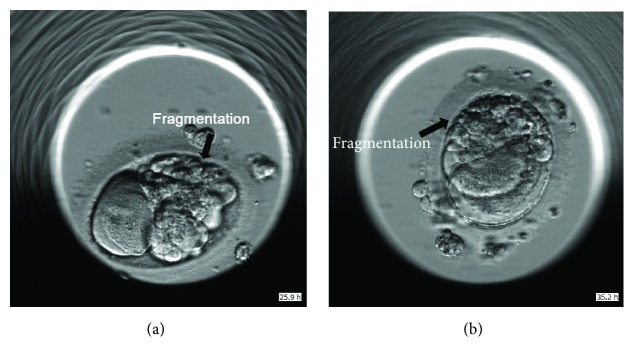
Two embryos after first cell division. The transferred embryo at 25.9 hours after insemination (a) and the embryo which was not transferred (b). Arrow shows fragmentations.

**Figure 2:**
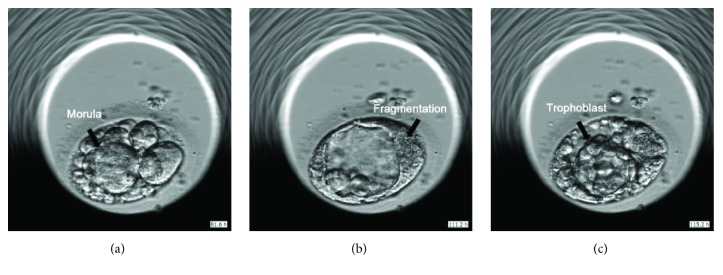
The transferred embryo from morula (81.6 hours) (a) to the blastocyst stage (115.2 hours), before (b) and after (c) collapse.

The patient was a thirty-year-old nulligravid woman, who underwent IVF treatment following 3 artificial inseminations by donor sperm (AID). In the first cycle, 17 oocytes were retrieved. Nine oocytes were matured and 5 of them were fertilized. Two fragmented embryos were obtained on day 2 and were transferred without achieving pregnancy. In the second cycle, 14 oocytes were retrieved. Twelve oocytes were matured and 10 of them were fertilized. As observed in the previous cycles, on day 2 all embryos were totally fragmented. None of them reached the blastocyst stage and no embryo transfer was performed. In the third cycle, 11 oocytes were retrieved, and 7 oocytes were matured and were fertilized. On day 2 all embryos were with more than 60% fragmentation except 2 which were estimated with about 40% fragmentation (Figure 1). One of these was divided into 2 cells at about 25 hours after insemination (Figure 1(a)) and the other around 35 hours after insemination (Figure 1(b)). Following a discussion with the patient, we continued monitoring the embryos until day 5, at which time 1 blastocyst was developed (Figure 1(a)). This embryo had the expected annotation (according to the producer model) with first division at 24.7 h and start blastulation at 114 h (Figure 2). This embryo collapsed and reexpanded. The score of this embryo was low because of the fragmentations, but in blastocyst stage, all fragments were pushed aside. This blastocyst was therefore transferred as a single embryo transfer. A healthy boy with birth weight 3500 gr was born at term.

## 4. Discussion

Several studies have shown the relation between number of cells on day 3, percentage of fragmentation and symmetry of the embryo, and live birth rate [7]. Fragmentation has been shown to be an important biomarker [8]: high fragmentation usually results in reduced cell volume and increased disorganization in the embryo [9]. Extensive fragmentation may be associated with reduced blastocyst formation. It can influence allocation of cells during differentiation and is associated with an increased incidence of chromosomal abnormalities [10, 11]. Embryo cell fragmentation frequently occurs as early as at the first embryo division. Approximately 40% of aneuploid embryos which develop to the blastocyst stage undergo self-correction [11]. We assume that this was the case in our patient but could only have verified self-correction if genetic analysis had been applied.

Different methods for embryo estimation and scoring have been proposed. Some of these methods based on proteomics, metabolomics, and more recently on small noncoding RNA, including microRNA, are too complex to perform routinely in the IVF lab [12].

Annotation of the fertilized oocytes in time-lapse is an applicable method for embryo selection [13]. This report presents the value of objective parameters in addition to the morphological estimation and the benefit of prolonged incubation of the IVF embryos. It is possible to assume that this embryo would have been transferred even without using EmbryoScope. However, the appropriate division kinetics annotation convinced us to continue embryo incubation until day 5 and then to transfer the embryo despite its poor morphologic feature. We suggest that prolonged embryo monitoring by time-lapse may be particularly valuable in patients in whom embryos are difficult to estimate regarding prognosis for achieving pregnancy and a live birth.
